# High-throughput SNP genotyping in the highly heterozygous genome of *Eucalyptus*: assay success, polymorphism and transferability across species

**DOI:** 10.1186/1471-2229-11-65

**Published:** 2011-04-14

**Authors:** Dario Grattapaglia, Orzenil B Silva-Junior, Matias Kirst, Bruno Marco de Lima, Danielle A Faria, Georgios J Pappas

**Affiliations:** 1 EMBRAPA Genetic Resources and Biotechnology - Estação Parque Biológico, final W5 norte, Brasilia, Brazil; 2 Genomic Sciences Program - Universidade Catolica de Brasília- SGAN, 916 modulo B, 70790-160 Brasília - DF, Brazil; 3 School of Forest Resources and Conservation, Genetics Institute, University of Florida, PO Box 110410, Gainesville, USA; 4 Department of Genetics - Universidade de São Paulo - ESALQ/USP - Av. Pádua Dias, 11 - Caixa Postal 9 13418-900 Piracicaba, SP, Brazil

## Abstract

**Background:**

High-throughput SNP genotyping has become an essential requirement for molecular breeding and population genomics studies in plant species. Large scale SNP developments have been reported for several mainstream crops. A growing interest now exists to expand the speed and resolution of genetic analysis to outbred species with highly heterozygous genomes. When nucleotide diversity is high, a refined diagnosis of the target SNP sequence context is needed to convert queried SNPs into high-quality genotypes using the Golden Gate Genotyping Technology (GGGT). This issue becomes exacerbated when attempting to transfer SNPs across species, a scarcely explored topic in plants, and likely to become significant for population genomics and inter specific breeding applications in less domesticated and less funded plant genera.

**Results:**

We have successfully developed the first set of 768 SNPs assayed by the GGGT for the highly heterozygous genome of *Eucalyptus *from a mixed Sanger/454 database with 1,164,695 ESTs and the preliminary 4.5X draft genome sequence for *E. grandis*. A systematic assessment of *in silico *SNP filtering requirements showed that stringent constraints on the SNP surrounding sequences have a significant impact on SNP genotyping performance and polymorphism. SNP assay success was high for the 288 SNPs selected with more rigorous *in silico *constraints; 93% of them provided high quality genotype calls and 71% of them were polymorphic in a diverse panel of 96 individuals of five different species.

SNP reliability was high across nine *Eucalyptus *species belonging to three sections within subgenus Symphomyrtus and still satisfactory across species of two additional subgenera, although polymorphism declined as phylogenetic distance increased.

**Conclusions:**

This study indicates that the GGGT performs well both within and across species of *Eucalyptus *notwithstanding its nucleotide diversity ≥2%. The development of a much larger array of informative SNPs across multiple *Eucalyptus *species is feasible, although strongly dependent on having a representative and sufficiently deep collection of sequences from many individuals of each target species. A higher density SNP platform will be instrumental to undertake genome-wide phylogenetic and population genomics studies and to implement molecular breeding by Genomic Selection in *Eucalyptus*.

## Background

High-throughput, high density SNP genotyping has become an essential tool for QTL mapping, association genetics, gene discovery, germplasm characterization, molecular breeding and population genomics studies in several crops and model plants [1-7]. The abundance of Single Nucleotide Polymorphisms (SNPs) in plant genomes together with the rapidly falling costs and increased accessibility of genotyping technologies, have prompted an increasing interest to develop panels of SNP markers to expand resolution and throughput of genetic analysis in less-domesticated plant species with uncharacterized genomes such as those of orphan crops [8], forest [9-12] and fruit trees [13-15].

Two main strategies have been employed to identify SNPs in plants: utilization of EST sequence information to direct targeted amplicon resequencing and, more recently, next generation sequencing (NGS) technologies coupled or not to genome complexity reduction methods [16]. Amplicon resequencing of stretches of target genes is carried out in a germplasm panel that is relevant to the downstream applications and sufficiently large to avoid ascertainment bias. SNPs are mined in the resulting sequences and then assays are designed focusing on those particular SNPs. This strategy, although labor intensive, has been successful when the goal is to develop a moderate number of assayable SNPs [16]. High throughput NGS and direct *in silico *SNP identification now provide a very effective alternative to amplicon resequencing for SNP development in plants [17]. Thousands of SNPs can be readily identified given that sequences are obtained from an adequately large representation of individuals with sufficiently redundant genome coverage. Complexity reduction strategies such as using cDNA libraries [18,19], AFLP derived representations [20], reduced representation libraries generated by restriction enzyme digestion and fragment selection [2,21], microarray-based [22] or in-solution [23] sequence capture, and additional target enrichment strategies [24] can be used to obtain the necessary sequence depth when the objective is to develop SNP based markers in specific genes or regions of the genome. Multiplexed bar-coded sequencing of such reduced genomic representations optimizes costs of SNP identification by increasing coverage and genotypic representation in the target regions [24-26]. Clearly the prospects are that sequence abundance and quality for SNP identification will no longer be a limiting factor for any plant genome.

A number of SNP genotyping technologies were developed in recent years mostly geared toward assaying human SNP variation. Among those that have been used in plant genetics, the Golden Gate Genotyping Technology (GGGT) developed by Illumina has consistently been reported as a reliable technology, displaying high levels of SNP conversion rate and reproducibility [16]. This assessment, initially reported for large scale human genotyping, has been corroborated in plant species including autogamous crops with low nucleotide diversity (0.2% to 0.5%) [3,27-29] and outbred species with much higher sequence diversity typically ≥2% [9-13]. In highly heterozygous genomes, the development of GGGT SNP assays has been carried out mainly by amplicon resequencing targeting specific genes. This approach has been practical in conifers using haploid megagametophyte tissue [30,31] and poplar for which a reference genome is available [12]. If attempted for large scale SNP development, however, this approach would be technically challenging for most outbred plant genomes due to the high levels of nucleotide diversity and additional indel variation as shown in earlier attempt for grape [32]. Direct SNP development from large *in silico *sequence resources will likely be the best approach for the highly heterozygous genomes of the majority of undomesticated plant species.

Irrespective of the method used to develop SNP markers in heterozygous genomes - direct *in silico *or targeted amplicon re-sequencing - challenges are faced in later steps when attempting to convert queried SNPs into high-quality genotypes. Particularly for the development of GGGT assays based on hybridization of allele and locus specific oligonucleotides, constraints have to be placed on the sequences flanking the target SNP [33]. A robust diagnosis of sequence variation in the vicinity of the target SNPs will depend largely on sequence coverage, sequence quality [34] and origin of sequences as far as the number and relatedness of individuals surveyed for SNP discovery. These issues will become increasingly exacerbated when attempting to transfer SNP assays across species within the same genus. Still a rarely explored topic in plants [13,30,35], the assessment of inter-specific transferability of SNPs will likely be an important subject for population genomics and inter specific breeding applications in less domesticated and less funded plant genera.

Species of *Eucalyptus *are currently planted in more than 90 countries and are well known for their fast growth, straight form, valuable wood properties and wide adaptability [36]. *Eucalyptus *subgenus *Symphyomyrtus*, includes the majority of the twenty or so commercially planted species. *E. globulus *has been the top choice for plantations in temperate regions. Tropical *Eucalyptus *forestry, on the other hand, is based on interspecific hybrid breeding and clonal propagation with *E. grandis *as the pivotal species [36]. Molecular marker technologies have allowed a significant progress in the genetics and breeding of this vast genus that includes over 700 species [36]. Genetic analyses with molecular markers were key to settle phylogenetic issues [37], manage breeding populations [38] build linkage maps [39-41] and identify QTLs for important traits [42-45]. Nonetheless, more extensive genome coverage, higher throughput and improved inter specific transferability of current genotyping methods are necessary to increase resolution and speed for a variety of applications. A DArT array delivering around 3,000 to 5,000 dominant markers for mapping and population analyses was recently reported [46]. SNP developments in species of the genus have targeted specific candidate genes generating a few tens SNPs for specific association genetics studies [47,48]. However, large scale SNP arrays developments for *Eucalyptus *are yet to come. Due to their recent domestication, large population sizes and outbred mating system, species of *Eucalyptus *are among the ones with the highest frequency of SNPs reported in woody plant species and possibly in plants in general, with up to 1 SNP every 16 bp [49]. While a bonus for overall SNPs identification, such high nucleotide diversity, both within and among species, could represent an obstacle for the development of large sets of robust and polymorphic sets of Golden Gate assayable SNPs across species.

We are interested in developing genome-wide parallelized genotyping methods to be used for the operational implementation of Genomic Selection in *Eucalyptus *hybrid breeding, population genomics and phylogenetic studies in natural populations of the genus. The upcoming availability of a reference genome for *Eucalyptus grandis *and the rapid evolution of high throughput sequencing technologies will foster the buildup of large sequence dataset from many individuals, a valuable resource for the development of large collections of SNPs for the genus. In anticipation to this time, we used a 1.2 million mixed EST dataset including Sanger and 454 sequences from multiple *Eucalyptus *species and individuals to: (1) develop and validate an initial collection of genome-wide SNPs for *Eucalyptus *derived exclusively from *in silico *EST sequence data from unrelated individuals of different species; (2) assess the effect of increasingly stringent *in silico *SNP identification and design parameters on the reliability and polymorphism of SNP genotyping in species of *Eucalyptus *using the Golden Gate Genotyping Technology (GGGT); (3) evaluate SNPs transferability across eleven species of *Eucalyptus *and polymorphism in the five main planted species worldwide. Information on all SNPs discovered and validated in the present study is provided.

## Results

### EST clustering, contig assembly and SNP discovery pipeline

**Figure 1:**
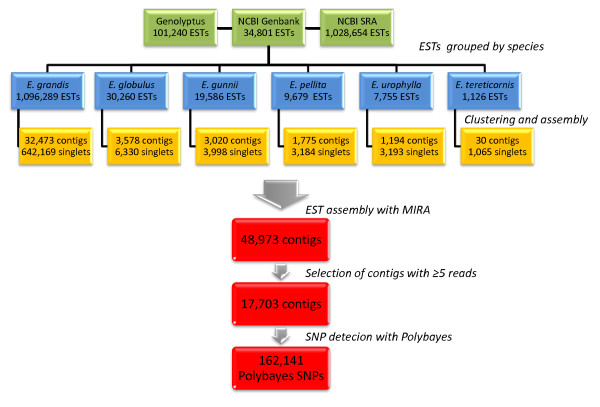
**Flowchart with the output results of the EST clustering, contig assembly and SNP discovery pipeline prior to applying SNP filtering and selection for the GGGT assay design**.

ESTs for six different species of *Eucalyptus *were used in this study to maximize the sampling of DNA sequence variation across species, although only a portion was retained for assembly after applying several quality filters. From a total of 136,041 Sanger-derived ESTs, 78,087 of them (57.4%) were further processed. Similar percentage was retained out of the 1,028,654 454-derived ESTs (60.7%) (Table 1). The majority of the Sanger reads and all 454 reads were obtained from *E. grandis*, the pivotal species in most tropical breeding programs, totaling 94% of the available ESTs before assembly and 96% after assembly, i.e. effectively used for SNP discovery. A two-step EST-assembly strategy was used: clustering performed at the species and sequencing technology levels followed by using the MIRA 2 assembler (Whole Genome Shotgun and EST Sequence Assembler) to consolidate the contigs and singletons from the previous step into a final EST assembly. After the MIRA assembly 48,973 contigs were obtained. Only those contigs formed by five or more ESTs were considered in this analysis to mitigate the limitations of alignment depth in SNP detection, thus resulting in 17,703 usable contigs (36.15% of the total). From this contig set, SNPs were predicted using the program PolyBayes. Only SNPs with high probability (P_SNP_≥0.99) were selected, totaling 162,141 potentially polymorphic sites (Figure 1).

**Table 1:** Summary of the EST assembly for SNP discovery

Sequencing technology	*Eucalyptus* species	# sequences used for clustering	# sequences in the assembly
Sanger	*E. grandis*	67,635	50,720
	*E. globulus*	30,260	10,088
	*E. urophylla*	7,755	4,387
	*E. gunnii*	19,586	7,018
	*E. pellita*	9,679	4,959
	*E. tereticornis*	1,126	1,095
454	*E. grandis*	1,028,654	623,922

TOTAL		1,164,695	702,009

### *In silico *selection of genome-wide SNP

Five sequential filters were applied to the 162,141 candidate genome-wide SNPs for GGGT assay design from F0 (less stringent) to F4 (most stringent) (see Methods). When the filtering stringency increased from F0 to F4, the number of SNPs surviving selection *in silico *decreased abruptly. A total of 66,254 SNPs (40.6%) were selected that had ≥ 5 reads on the SNP position and a minimum of one read with the alternative base. This number dropped to 21,944 (13.5%) when an *in silico *MAF ≥ 0.2 constraint was applied and to 10,032 (6.2%) when at least one EST from the more distant species *E. globulus *or *E. gunnii *was required in the contig. When the filter requiring flanking sequence conservation was applied, the number of SNPs selected dropped even further to a final number of only 1,329 when a cutoff of 60 bases with no additional SNP on each side of the target SNP was stipulated. The number of unigene contigs retained along the filters also dropped significantly from an initial number of 17,703 to a mere 998 when all filtering constraints were applied (Table 2). Overall the proportion of SNPs with ADT (Assay design Tool) score greater than 0.6, i.e. SNPs with a high likelihood to be converted into a successful genotyping assay, was around 95%, irrespective of the filtering treatments. For example, by applying only filter F0, 598 SNPs out of 621 had ADT score ≥ 0.6; similarly, with filter F4, 525 out of 547 SNPs had ADT score ≥ 0.6. The proportion of SNPs with ADT score ≥ 0.9 was between 50 and 53% again showing no impact of the filtering treatments (Table 2). For bench validation only SNPs with ADT score ≥ 0.8 were selected. A list of the 696 genome-wide SNPs selected and tested by the Golden Gate assay is available in Additional file 1.

**Table 2:** Summary of the *in silico *SNP development procedure using increasingly stringent SNP selection and design requirements (F0 through F4) (see methods for details)

In silico SNP performance assessment	F0	F1	F2	F3	F4
**# of SNPs**	66,254	21,944	10,032	3,187	1,329
**# of contigs with SNPs**	9,579	5,058	2,057	1,651	998
**# of SNPs submitted to the ADT**	621	605	583	367	547
**# of SNPs with ADT Score **≥ **0.6**	598	572	557	353	525
**% of SNPs with ADT Score **≥ **0.6**	96.3	94.5	95.5	96.2	96.0
**# of SNPs with ADT Score **≥ **0.9**	314	316	297	177	291
**% of SNPs with ADT Score **≥ **0.9**	50.6	52.2	50.9	48.2	53.2
**# of SNPs tested by the GGGT**	96	96	108	108	288

### SNP discovery in pre-determined candidate genes

From a list of 42 candidate genes selected from the literature as being putatively associated with relevant wood phenotypes in *Eucalyptus *(see Material and Methods), only in 20 of them SNPs were found that matched the minimum requirements of having ≥ 2 reads with alternative bases at the SNP position and at least 60 bases of flanking sequence on each SNP side. For these 20 genes, a total of 175 SNPs were discovered and 72 were included in the bead array for downstream validation. These 72 SNPs were selected to assay at least one SNP in each one of the 20 genes and in those genes where several SNPs were available, SNPs that were derived from a contig with at least one read coming from *E. globulus *or *E. gunnii *and distantly positioned along the contig were selected. These 72 SNPs assayed in candidate genes are available as a separate spreadsheet in Additional file 1.

### SNP genotyping reliability

**Figure 2:**
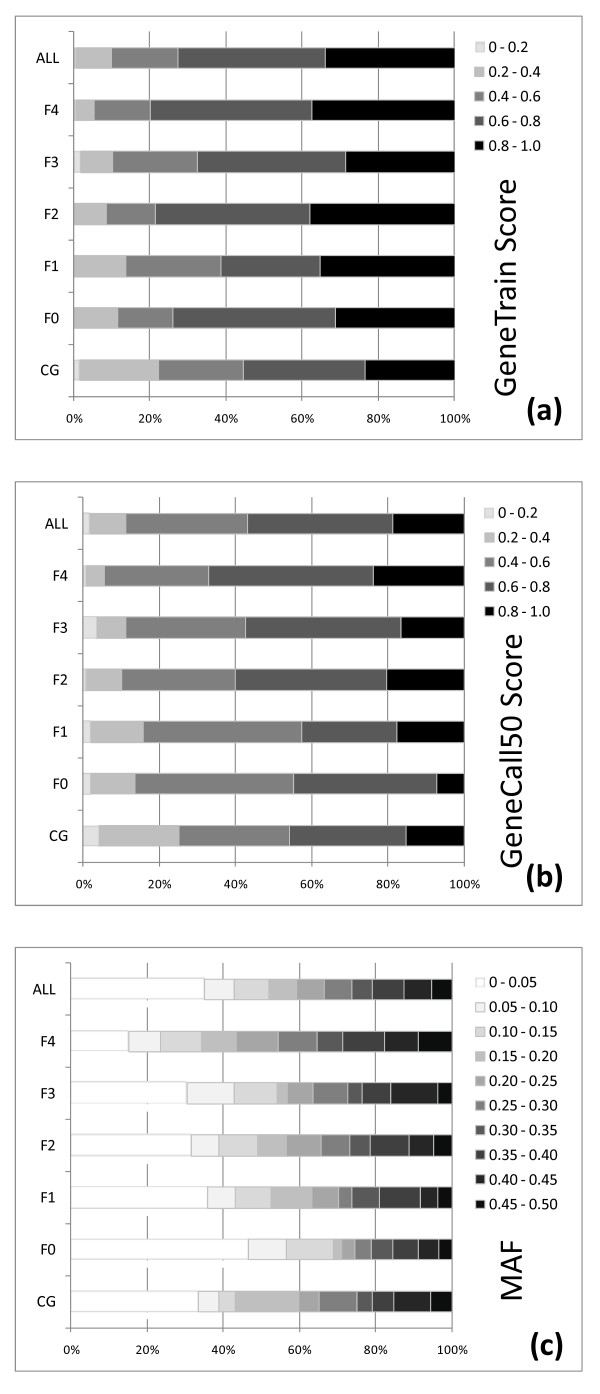
**Distribution of the percentages of SNPs across classes of (a) GeneTrain Score; (b) GeneCall50 Score and (c) Minimum Allele Frequency (MAF) **. Broken bars histograms are presented for all 768 SNPs together (ALL) and for each SNP category within the 696 genome-wide SNPs selected by the different *in silico *filtering levels (F0 through F4 - see methods) and the 72 candidate gene (CG) SNPs.

The distributions of the proportions of SNPs in increasingly more reliable classes as measured by the GeneCall50 and GeneTrain scores for each *in silico *filter level were plotted (Figure 2). The relative distribution of the broken bars histograms corresponding to increasing levels of reliability suggests that when progressively more stringent *in silico *SNP selection requirements are applied from F0 to F4, larger proportions of SNPs with higher GeneTrain and GC50 scores were obtained. For SNPs in pre-determined candidate genes (CG) the proportions of SNPs at the lower ends of the distribution of GC50 and GeneTrain scores were larger reflecting the less stringent *in silico *selection applied in these cases (Figure 2). SNPs developed in specific candidate genes for which limitations existed regarding the number of available EST reads, generally showed a slightly lower performance in all measured parameters of reliability even when compared to SNPs developed only applying filter F0. The proportion of SNPs with call rate rate ≥ 95% was only 80.6%, the average GeneTrain score was the lowest at 0.61, and the proportion of SNPs with GeneTrain and GC50 scores ≥ 0.40 was less than 90%. However no difference was seen in the proportion of polymorphic SNPs in relation to the more stringent *in silico *filtering levels. Because SNPs in candidate genes were mined without observance of any specific *in silico *filtering level besides the most fundamental one (see methods), they were not included in the subsequent comparative analyses of the *in silico *filtering parameters.

The overall genotyping reliability for the 768 SNPs was assessed by estimating SNP counts above conventionally used threshold and average values for Call Rate, GeneCall and GeneTrain scores (Table 3). Goodness-of-fit for normality tests showed that all these three variables were not normally distributed (p < 0.0001). The average call rates for all SNPs, irrespective of *in silico *filter levels were above 90%; 87% of all 768 SNPs had call rates ≥ 95%. Mann-Whitney non-parametric tests showed no significant difference in average call rate and GeneTrain score between filtering levels tested individually or combined based on requirements of conservation of flanking sequences (F0+F1+F2 against F3+F4). The proportion of SNPs with call rates ≥ 95% varied, with an increasing trend when going toward a more stringent SNP filtering selection and reaching 93.1% with filter F4. When tested pair-wise and sequentially, i.e. F0 against F1, F1 against F2 and so on, no significant differences in the proportion of SNPs with call rates ≥ 95% or GeneTrain ≥ 0.4 were found using a Chi-square Pearson test. However when the pooled count of all SNPs selected with no requirements of conservation of flanking sequences (filters F0+F1+F2; 245 in 300) was compared to the count of SNPs selected with such requirements (i.e. no additional SNPs either in 20 or 60 bases on each SNP side, i.e. filters F3+F4; 365 SNPs in 396) (Table 3), a highly significant difference was found in the final number of SNPs recovered with call rates ≥ 95%, (Chi-square Pearson = 17.40; p = 0.00003). SNP reliability based on the GeneCall50 score followed a similar trend observed with the Call Rate and GeneTrain with an increase from 0.59 for F0 to 0.67 for F4. However a significant difference in the average GC50 score was found when the comparison was between the pooled SNPs from filters F0+F1+F2 (GC50 = 0.61) and those derived from filters F3+F4 (GC50 = 0.66) (Mann-Whitney non-parametric test p = 0.000041). These results indicate that although the vast majority of SNPs could be robustly scored with high call rate, a more stringent *in silico *selection on the flanking sequences yields more SNPs with higher call rates and GeneTrain scores as well as SNPs with average higher GeneCall50 scores. We used a relatively stringent GeneCall50 cutoff of 0.4 when compared to other SNP development studies as we observed that at lower thresholds, the genotype cluster separation consistently showed undesirable shifts.

**Table 3:** Summary of the in vitro SNP genotyping performance assessed in a panel of 96 individuals from five *Eucalyptus *species

In vitro SNP performance assessed	Candidate genes	F0	F1	F2	F3	F4	Total counts	%
# SNPs tested by the GGGT	72	96	96	108	108	288	768	-
Average SNP Call Rate (%)	91.0	95.2	90.0	94.9	95.0	97.8	-	
# SNP with Call rate ≥ 0.95	58	81	74	90	97	268	668	87.0
% SNP with Call rate ≥ 0.95	80.6	84.4	77.1	83.3	89.8	93.1	-	
Average SNP GeneTrain score	0.61	0.68	0.66	0.71	0.67	0.72	-	
# SNPs with GeneTrain score ≥ 0.40	64	90	90	100	101	278	723	94.1
% SNPs with GeneTrain score ≥ 0.40	88.9	93.8	93.8	92.6	93.5	96.5	-	
Average SNP GC50 score	0.57	0.59	0.59	0.64	0.62	0.67	-	
# SNPs with GC50 score ≥ 0.40	63	89	89	100	101	277	719	93.6
% SNPs with GC50 score ≥ 0.40	87.5	92.7	92.7	92.6	93.5	96.2	-	
Average MAF of SNPs with MAF ≥ 0.05	0.26	0.24	0.25	0.26	0.25	0.27	-	
# SNP with MAF > 0.05	51	48	55	75	74	205	508	66.1
% SNP with MAF > 0.05	70.8	50.0	57.3	69.4	68.5	71.2	-	

Averages and SNP counts above specific thresholds of SNP reliability parameters (Call Rate, GeneCall50, GeneTrain scores) and polymorphism (MAF) for SNPs in pre-selected candidate genes and for genome-wide SNPs selected with increasingly stringent *in silico *SNP selection and design requirements (F0 through F4 - see methods for details).

### SNP polymorphism

The proportion of polymorphic SNPs overall the five main *Eucalyptus *species (N = 96 individuals) for all 768 SNPs was estimated at 66.1%, which corresponds to the conversion rate. When only the 711 SNPs that simultaneously met the *adhoc *thresholds of reliability (GC50 ≥ 0.4 and Call Rate ≥ 95%) are considered, a higher proportion of them are polymorphic with MAF ≥0.05 (505 in 711) i.e. a conversion rate of 71%. The average MAF of polymorphic SNPs was consistently around 0.25 for all filtering levels and for the candidate gene SNPs as well (Table 3). The proportion of SNPs with higher polymorphism level, measured by MAF, increased as progressively more stringent selection was applied *in silico *as depicted in the broken bars histogram. However only with the more rigorous F4 selection on the SNP flanking sequence a larger proportion of polymorphic SNPs was effectively recovered (Figure 2). No increase was seen in the proportion of polymorphic SNPs when going from filter F1 (69.4%) to F2 (68.5%), i.e. by including the requirement of ESTs reads from section Maidenaria in the contig (Table 3). However the proportion of polymorphic SNPs significantly increased from selection with filters F0+F1+F2 (175 in 300) to selection with filters F3+F4 (279 in 396) (Chi-square Pearson = 9.36; p = 0.00221), suggesting that the inclusion of a filtering requirement on the SNP flanking sequences not only results in more reliably assayable SNPs but also increases the proportion of polymorphic SNPs.

The proportions of polymorphic SNPs were also estimated for each main species separately, and for all possible combinations of species, i.e. the number of SNPs that were polymorphic for the species simultaneously (Table 4 and Additional file 2). In this analysis only the 711 SNPs that simultaneously met the *adhoc *thresholds of reliability were considered. The highest proportions of polymorphic SNPs were observed for *E. grandis, E. urophylla *and *E. camaldulensis*, between 40.9% and 49.4%, while in the two species of the more distant section Maidenaria, the proportion of polymorphic SNPs was around 22 to 25%. The average number of polymorphic SNPs in all three-way species combinations varied from a maximum of 144 (20%) for the *E. grandis, E. urophylla *and *E. camaldulensis *set to a minimum of 77 (11%) for the *E. urophylla, E. globulus *and *E. camaldulensis *set. Only between 64 and 78 SNPs were polymorphic when any four species combinations were considered and only 55 (7.7%) when all five were taken into account (Additional file 2). Given the relatively limited sample size, when a less conservative estimate of polymorphism within species was used (MAF ≥ 0.01) the proportions of polymorphic SNPs increased considerably in all species and combinations. For example in *E. grandis *the proportion went from 49.4% to 62%, in *E. camaldulensis *from 41.2% to 58.5% and in *E. globulus *from 22.2% to 33.6%. Likewise SNPs that were polymorphic in two or more species concurrently also increased.

**Table 4:** Counts and percentages of polymorphic SNPs (MAF ≥ 0.05) from a total of 711 reliable SNPs, in each one of the five main *Eucalyptus *species surveyed (diagonal) and in pair-wise sets of species (above the diagonal)

	*E. grandis*	*E. urophylla*	*E. globulus*	*E. nitens*	*E. camaldulensis*
*E. grandis*	**351 (49.4%)**	209 (29.4%)	117 (16.5%)	128 (18.0%)	194 (27.3%)
*E. urophylla*		**291 (40.9%)**	107 (15.0%)	120 (16.9%)	187 (26.3%)
*E. globulus*			**158 (22.2%)**	104 (14.6%)	118 (16.6%)
*E. nitens*				**181 (25.5%)**	127 (17.9%)
*E. camaldulensis*					**293 (41.2%)**

### SNP reliability across subgenera

Based on the results showing a significant increase in SNP genotyping reliability when introducing *in silico *constraints on SNP flanking sequences, SNP reliability across a larger set of species and subgenera was evaluated by considering only two overall SNP selection levels: (1) SNPs selected with no requirement of conservation of flanking sequences (this group includes candidate genes SNPs plus genome-wide SNPs from filters F0+F1+F2, totaling 372 SNPs) and (2) SNPs selected requiring conservation in flanking sequences of either 20 or 60 bases (this group includes genome-wide SNPs from filters F3+F4 with a total of 396 SNPs). Reliability was assessed by the counts and proportion of SNPs that displayed a Call rate ≥ 0.95 and a GC50 score ≥ 0.40 (Table 5). A comparison of the GeneTrain score across species does not apply in this case, as it is a SNP specific statistics appraising the quality of the genotype clusters and remains unchanged for all samples used to generate the clusters. The relative proportions of reliable SNPs across all nine species of subgenus Symphyomyrtus did not vary much within each SNP selection level. With no flanking sequence constraints on average 81% of the SNPs had call rate ≥ 0.95 and 88% a GC50 score ≥ 0.40. With flanking sequence constraints the proportions were higher, 90% of the SNPs had call rate ≥ 0.95 and 94% a GC50 score ≥ 0.40. However, a lower genotyping reliability was observed for the two species outside subgenus Symphyomyrtus, with only around 50% of the SNPs having satisfactory call rate and GC50 scores even for SNPs selected with flanking sequence constraints. In all eleven species but *E. cloeziana*, a significant increase was found (Pearson chi square test p <0.01) in the number of SNPs that met or exceeded the call rate and GC50 thresholds when flanking sequence constraints were applied *in silico *(Table 5). This result confirms the impact of flanking sequence constraints on the reliability of SNPs in all tested species, irrespective of the presence of ESTs from the particular species in the database used for SNP discovery.

**Table 5:** Summary of SNP reliability across species, sections and subgenera of Eucalyptus as measured by the number of SNP meeting the thresholds of call rate and GeneCall50 for two groups of SNPs that differed regarding the flanking sequence constraints during *in silico *SNP mining and GGGT assay design

		SNPs selected with no flanking sequence requirements (N = 372)				SNPs selected with no additional SNPs in flanking sequence (N = 396)			
Subgenera/Section	Species	# SNPs with Call rate	% SNPs with Call rate	# SNPs with GC50	% SNPs with GC50	# SNPs with Call rate	% SNPs with Call rate	# SNPs with GC50	% SNPs with GC50
		≥ 95%	≥ 95%	≥ 0.40	≥ 0.40	≥ 95%	≥ 95%	≥ 0.40	≥ 0.40
*Symphyomyrtus/Latoangulatae*	*E. grandis*	323	86.8	333	89.5	378	95.5	378	95.5
*Symphyomyrtus/Latoangulatae*	*E. urophylla*	310	83.3	335	90.1	369	93.2	377	95.2
*Symphyomyrtus/Latoangulatae*	*E. saligna*	279	75.0	328	88.2	343	86.6	376	94.9
*Symphyomyrtus/Maidenaria*	*E. globulus*	325	87.4	331	89.0	369	93.2	374	94.4
*Symphyomyrtus/Maidenaria*	*E. nitens*	311	83.6	327	87.9	369	93.2	375	94.7
*Symphyomyrtus/Maidenaria*	*E. dunnii*	295	79.3	324	87.1	361	91.2	371	93.7
*Symphyomyrtus/Maidenaria*	*E. viminalis*	300	80.6	325	87.4	353	89.1	370	93.4
*Symphyomyrtus/Exsertaria*	*E. camaldulensis*	289	77.7	336	90.3	339	85.6	376	94.9
*Symphyomyrtus/Exsertaria*	*E. tereticornis*	281	75.5	319	85.8	330	83.3	365	92.2
*Eucalyptus/Pseudophloius*	*E. pilularis*	194	52.2	271	72.8	246	62.1	325	82.1
*Idiogenes/Gympiaria*	*E. cloeziana*	166	44.6	223	59.9	198	50.0	278	70.2

### Heritability-based SNP validation

SNP assay quality was further assessed by estimating heritability of allelic transmission in parent-parent-offspring trios involving different *Eucalyptus *species as parents. Heritability is defined as the number of offspring genotypes that agree with the expected inheritance over the total number of genotype calls possible. In family *E. grandis × E. urophylla *(G × U) there were 457 Mendelian transmission inconsistencies out of the 36,864 allelic transmissions assayed, i.e. a genotyping miscall rate of 1.2%. In total 719 SNPs out of the 768 tested (93.6%) had 100% heritability and 80% of the inheritance miscalls were concentrated in 24 SNPs. In the four species family ([*E. dunni × E. grandis] *× [*E. urophylla × E. globulus]*) (DG × UGL) 1,596 transmission inconsistencies were seen, i.e. a genotyping miscall rate of 4.3%, only 678 SNPs (88.3%) had 100% heritability and 80% of the inheritance miscalls were concentrated in 71 SNPs. Only 17 SNPs displayed miscalls in both families concurrently, revealing potentially more problematic SNPs. Upon inspection of the SNPs clustering graphs most inheritance miscalls in both families were due to the two parents being homozygous AA and BB and offspring not having the expected genotype AB but rather one of the two homozygous ones.

### Sequence-based validation of SNP genotypes

SNP validation was possible for 50 SNPs for which five or more genomic reads overlapping at the SNP position with sequence quality Q ≥ 20 were obtained. Given the limited sample size available (number of observed reads at the SNP site) a less conservative alpha level α = 0.1 was used to increase the power of the binomial test used to declare sequence-based genotypes. In other words, by increasing the chance of obtaining a statistically significant result, the probability of correctly declaring a sequence-based homozygous genotype in spite of the small number of observed reads was increased although at the expense of an increase in Type I error, i.e. erroneously declaring the genotype as homozygous when in fact it is heterozygous. Sequence-based genotypes at 43 of the 50 SNPs (86%) matched the Golden Gate assay called genotypes (Additional file 3).

## Discussion

We have successfully developed the first set of 768 SNPs assayed by the Golden Gate genotyping technology for the highly heterozygous genome of *Eucalyptus*. The overall SNP success rate was high, with 87% of all SNPs showing call rates ≥ 95%, 94.1% of them having a GeneTrain score ≥ 0.40 and 93.6% a GeneCall50 score ≥ 0.40. The conversion rate, which is the proportion of polymorphic SNPs divided by the total number of SNPs was 66.1% estimated in a diverse panel of 96 individuals of five different species (Table 3). These are the first results of a larger scale SNP development effort for *Eucalyptus *suggesting that the Golden Gate assay performs well both within and across species notwithstanding the high nucleotide diversity of the complex *Eucalyptus *genome and the wide range of species for which SNP genotyping is pursued.

### SNP discovery and selection from *Eucalyptus *ESTs

SNP discovery and assay development was carried out based on all available 1,164,695 ESTs in public and our own databases as of May 2009 (Table 1). Although this was considered a large EST set by pre-next-generation sequencing standards, it constitutes a relatively small collection given today's sequencing technologies. A large number (162,141) of potentially polymorphic sites was found after EST clustering and assembly in agreement with the previous abundance of SNPs reported for species of *Eucalyptus *from *in silico *surveys [18,49]. However only 36% of the assembled contigs met the depth requirement of five reads overlapping the SNP position with 60 bases of available sequence on each side recommended for Golden Gate genotyping (Figure 1). In fact when SNPs were searched in 42 pre-determined candidate genes of interest, only 20 of them were available for SNP assay design. This result suggests that if SNPs are to be developed for specific genes from direct *in silico *sequence resources, a much higher sequence coverage than the one used in this work is necessary. Recently, such an approach proved successful by massive sequencing of reduced representation libraries of multiple grape varieties to develop a ~9,000 selected SNP array from over 470,000 *in silico *detected SNPs [13]. Several genetically heterogeneous plant genomes should be amenable to this same SNP development approach opening concrete perspectives for high throughput genotyping in a large number of less characterized, largely undomesticated species.

### SNP reliability is enhanced by stringent *in silico *constraints

Knowledge of the SNP flanking sequences is an important aspect of the success of the Golden Gate assay. The assay design tool provided by Illumina checks for the presence of repetitive or palindromic sequences, GC content and neighboring polymorphisms to provide a functionality score for each candidate SNP [33]. However no systematic assessment of the impact of additional polymorphisms in the flanking sequence of the target SNP on its genotyping reliability has been reported. While this represents a minor concern for species of low nucleotide diversity such as humans, crop plants and domestic animals, it is a key issue for highly heterozygous genomes with nucleotide diversity in excess of 1%. In the heterogeneous genome of loblolly pine, for example, Eckert et al. [9] suggested that the SNP success rate observed (67%), lower than the typical ≥ 90% rate obtained in crop plants and humans, could be attributed to the presence of undetected SNPs in the flanking sequences, but no detailed assessment of this issue was carried out. In spruce, no specific selection for conserved flanking sequences was carried out during SNP development; SNP success rates were around 69 to 77% [11]. In *Pinus pinaster*, the proportion of successful SNPs (GeneTrain > 0.25) developed from *in silico *was estimated at 61.5% while for SNPs developed by targeted amplicon resequencing it was slightly higher, at 73% but also no specific selection for more conserved SNP flanking sequences was carried out [10].

In our study we used five sequential *in silico *filters on the initial set of 162,141 candidate genome-wide SNPs discovered in 17,703 EST contigs that had ≥ 5 reads. While filter F0 was a commonly used criterion for SNP discovery *in silico*, F1 added a requirement for a minimum *in silico *estimated MAF ≥ 0.2. This single additional requirement, however, reduced to less than 1/3 the number of available SNPs for assay design (Table 2). Filter F2 introduced a requirement of inter-specific sequence representation in the contig to increase sequence sampling both at the SNP position as well as for flanking sequences, in an attempt to increase SNP transferability across more distant species. This further filter caused a reduction of 50% in the number of available SNPs. When filters F3 and F4 added a progressively more rigorous requirement on the SNP flanking sequences, the number of surviving SNPs decreased rapidly to a point that only 3,187 SNPs in 1,651 genes remained for SNP assay design after filter F3 or 1,329 SNPs in 998 genes after F4 (Table 2). The application of similarly stringent *in silico *quality filters to the initial SNP source also caused a 10-fold reduction in the available putative SNP when developing a 54,000 SNP array for bovine, but resulted in an increase from 50% to >85% in the conversion rate [50]. In our study, however, it is important to note that the observed reduction in the number of available SNPs was largely a result of the relatively limited number of ESTs available at the beginning of the pipeline (702,009), many derived from short 454 reads, so that the minimum *in silico *MAF ≥ 0.2 and sufficient flanking sequences could not be achieved in most contigs. Additionally only ~17,000 ESTs from section Maidenaria (*E. globulus *plus *E. gunnii*) were available among the 702,009 used (only 2.4%), strongly limiting the ability to fulfill the requirement of filter F2. This highly unbalanced sequence representation most likely was responsible for this sharp decrease in sequences used for SNP assay design. Had we had access to a more balanced EST representation across species, a much larger number of SNPs would probably have survived all sequential filters and be amenable to assay design.

Our results show that the increasingly more stringent requirements on the SNP surrounding sequences are highly effective and have a statistically significant impact not only on SNP reliability but also on the proportion of polymorphic SNPs. Significantly more SNPs with higher call rates and GenCall50 scores were observed (p < 0.001) when filters F3 and F4 on flanking sequences were applied (Table 3). Furthermore, although comparison of SNP success rates across studies is not clear-cut due to the peculiarities of SNPs discovery and SNP reliability thresholds used, our overall SNP success rate averaged 87% if measured by the percentage of SNP with call rate ≥ 95%, or 94% if measured by the proportion of SNPs with GeneTrain and GeneCall50 ≥ 0.4 (Table 3). For the 288 SNPs selected with the most stringent filtering level F4, over 96% of them had GeneTrain and GeneCall50 ≥ 0.4. These assay success rates are comparable to those obtained for the human [33] and barley [3] genomes. It is worth mentioning, however, that our considerably higher success rates when compared to other studies with highly heterozygous tree genomes, likely derives from the fact that the vast majority of the ESTs used were obtained from a relatively large sample with more than 21 unrelated diploid individuals (i.e. more than 42 sampled chromosomes) of *E. grandis*. More importantly, the pipeline filtered out SNPs that did not belong to the same exon by using the draft genome sequence for *E. grandis*, therefore avoiding failures due to SNP located in intron/exon junctions, a considerable drawback when developing SNPs from ESTs [51]. The impact of using a reference genome was likely responsible for the comparably high success rate ≥ 87% for the candidate genes SNPs for which no flanking sequence requirements could be applied. In summary, although we did not compare the reliability of SNPs designed without using a final selection step based on the reference genome, the simple comparison of our success rates with those obtained for comparably heterozygous tree species supports the value of having access to a reference genome sequence for successful large scale SNP development.

### SNP conversion rate was increased by selecting for conserved SNP flanking sequences

An overall conversion rate of 66.1% was observed when genotype data for all 768 SNPs in a panel of 96 individuals of five species was considered. If only the 711 reliable SNPs are considered, the conversion rate increases to 71% which corresponds to the conversion rate of the top 288 SNPs developed after applying filter F4 on the SNP flanking sequences (Table 3). This conversion rate is equivalent to the one obtained for catfish SNPs developed from *in silico *ESTs after applying constraints on the number of ESTs and on the presence of minor allele sequences in the contig [51], and slightly higher than the conversion rates obtained for SNPs developed from *in silico *resources with no stringent filtering and assayed in analogous population samples of *Pinus pinaster *[10]. Interestingly, the proportion of polymorphic SNPs significantly increased (p = 0.00221) when flanking sequence conservation of 60 bases was required. We hypothesize that the effect of flanking sequence conservation on polymorphism is not a direct one. It is partly a result of the higher SNP reliability but probably also due to an indirect effect of assaying a SNP surrounded by higher quality flanking sequences likely devoid of sequencing errors, and thus selected as more conserved. Such a SNP is therefore less likely to be a false SNP due to sequencing errors in one or more of the reads in the contig resulting in a better *in silico *assessment of polymorphism and consequently a more polymorphic one when assayed at the population level.

### Estimates of polymorphic SNPs within *Eucalyptus *species are conservative

SNP polymorphism levels were also estimated for five species independently for which samples between 16 and 24 individuals (32 or 48 alleles) were genotyped (Table 4). The highest estimate was obtained for *E. grandis *(49.4%) followed by *E. camaldulensis *(41.2%) and *E. urophylla *(40.9%). These estimates are relatively low when compared to other SNP development studies in forest trees especially bearing in mind the high nucleotide diversity in *Eucalyptus*. Estimates of MAF in SNP development studies are, however, strongly influenced by the sample size and by the genetic origin of the population [10]. For example, a sample size of 146 individuals (292 alleles) would be necessary to estimate an allele with frequency 0.05 ± 0.025 with 95% probability. The samples sizes used in our study were therefore not optimal to detect low frequency alleles at several SNPs that would otherwise be deemed polymorphic had we used a larger sample size. Furthermore, none of the individuals used to generate the ESTs were present in the genotyped panel. In fact several species were not even represented in the EST databases such as *E. nitens *and *E. camaldulensis *and even for *E. globulus and E. urophylla *the proportion of sequences used was very limited, less than 2% and 1% respectively. Therefore the estimates of the proportion of polymorphic SNPs in each species individually are conservative and should be taken as a lower bound estimate. Conversion rates will likely improve considerably by selecting SNPs from a sequence database built from a much wider representation of the diversity of each target species and validating in a larger panel of individuals.

As expected, the highest rate of polymorphic SNPs was observed for *E. grandis*, the predominant species in the EST database with over 96% of the sequences used for SNP discovery. Interestingly, however, *E. camaldulensis *showed the second highest conversion rate (41.2%) despite the fact that not a single sequence was used for SNP discovery and that only 16 individuals, as compared to 24 in *E. grandis*, were genotyped. This result could be explained by a recent study that found *E. camaldulensis *with the highest nucleotide diversity among four *Eucalyptus *species, estimated at 1 SNP every 16 bp when amplicons in 23 genes were resequenced in 456 individuals from 93 populations [49]. In that same study several hundred individuals of *E. globulus *and *E. nitens *were also surveyed showing much lower nucleotide diversity, 31 and 33% respectively, in an equivalently wide sample of individuals and populations. In our study these two species displayed the lowest proportion of polymorphic SNPs (22.2 and 25.5%) (Table 4) and no statistically significant effect on the recovery of polymorphic SNPs was obtained by including at least one read from Maidenaria species (*E. globulus *or *E. gunnii*) in the contig, i.e. going from filter F1 to F2. Besides the ascertainment bias due to the very limited or nil representation of sequences in the EST databases, the lower proportion of polymorphic SNPs observed in these two species could be explained not only by their greater phylogenetic distance from *E. grandis *as compared to *E. urophylla *and *E. camaldulensis *but also by their intrinsically lower nucleotide diversity.

A substantial reduction in the proportion of simultaneously polymorphic SNP in two or more species was observed. The highest proportion of shared polymorphic SNPs was seen for the two and three-way combinations of *E. grandis, E. urophylla *and *E. camaldulensis *which agrees with their closest phylogenetic relationship. When Maidenaria species were included, however, the proportion of shared polymorphic SNPs dropped considerably to 14 to 18% and to 7.7% when all five species were contemplated together (Table 4 and Additional file 2). These results are consistent with *in silico *SNP sharing rates among four *Eucalyptus *species, estimated between 20 and 43% for 23 resequenced genes in much larger sample sizes [49]. In spite of the ascertainment bias that both *in silico *and assay-based estimates of shared polymorphic SNP suffer, these proportions suggest that a large number SNP pre-dating species separation will be available for assay development. From the practical standpoint this means that it is possible to develop a SNP array with informative SNPs across multiple *Eucalyptus *species. However the success of such an effort will strongly depend on their phylogenetic relationship and an extensive sampling of genome sequences of numerous individuals of each species. Furthermore all SNPs developed in our study were derived from expressed sequences, including 5' untranslated regions and exons. Kulheim et al. [49] showed a considerably higher SNP variability in introns when compared to exons in 23 genes in four *Eucalyptus *species. This result suggests that a higher SNP conversion rate could possibly be obtained in future SNP development efforts by screening SNPs derived from genomic sequences generated by massive NGS. On the other hand, however, the highest polymorphism of intronic and intergenic sequences should render more challenging the selection of SNPs with flanking sequences with no additional SNPs.

### SNP genotype calls match sequencing data and are correctly inherited in inter-specific crosses

SNP validation was carried out two-ways: by parent-parent-offspring allele transmission test in two unrelated pedigrees and by shallow next-generation sequencing of a single individual of *E. camaldulensis*. The inheritance assessment showed an overall high rate of correct Mendelian transmission with almost 99% of correct genotype calls in the *E. grandis *× *E. urophylla *pedigree and above 95% in the more diverse four-species pedigree. Reported genotyping inheritance miscall rates with the GGGT assay have been essentially zero in humans [33] and rarely reported for non-model plant genomes. Recently however, a global genotyping error rate of 0.54% in 188 SNPs was reported for *Pinus pinaster *[10] and between zero and 1% in polyploid wheat [52]. While the genotyping miscall rate of 1.2% in the *E. grandis × E. urophylla *pedigree falls within expectations, the much higher 4.3% rate in the four-species family is probably a result of a reduced SNPs transferability to this more diverse genomic background. Alternatively these higher miscall rates could be due to paralogous genomic sequences being assayed, although generally this has not been a major problem with the GGGT even in complex plant genomes [27]. We decided to use inter-specific pedigrees for a rigorous SNP inheritance assessment considering that several envisaged application of SNP genotyping in *Eucalyptus *will contemplate progenies from wide inter-specific crosses both for QTL mapping and the implementation of Genomic Selection. In both of these applications, however, a low proportion of genotype miscalls can be tolerated. This multi-species inheritance validation should be useful to reveal error-prone SNPs providing an additional selection criterion when developing a larger set of SNPs for genus wide genotyping in *Eucalyptus*.

Concordance between GGGT called genotypes and short-read sequencing was 86%. This NGS-based SNP validation approach is practical, especially in highly heterozygous genomes where direct amplicon sequencing can be challenging, but evidently has some limitations as SNP sampling is strongly dependent on sequence coverage. Five divergent genotypes were called homozygous by the GGGT assay and inferred as heterozygous by sequencing. A manual curation of these discordant cases suggested that these could be due to paralogous genes being sequenced, although they could also be caused by sequencing errors. Two SNPs called as heterozygous by the GGGT but homozygous by sequencing correspond to false positives possibly due to the small number of reads available, only five for one SNP and six for the other (Additional file 3).

### SNP detection with MIRA could significantly enhance SNP conversion rates

In an attempt to establish useful *in silico *predictors to guide future SNP development efforts, we further investigated the impact of the *in silico *variables used in the pipeline on SNP reliability measured by the GeneCall50 score and polymorphism by the MAF in *E. grandis *for which a large number of EST sequences was available. These variables were: 1) *in silico *estimated MAF; 2) the number of EST reads of a species at the SNP site; and 3) minimal distance to the next SNP site. GeneCall50 and MAF were modeled as binary response variables based on the established thresholds, i.e. SNPs were considered reliable if GeneCall50 ≥ 0.4 and unreliable otherwise and polymorphic if MAF ≥ 0.05 and monomorphic otherwise. Data were analyzed by means of a logistic regression [53] using each one of the explanatory variables above. The criterion for considering a SNP as polymorphic *in silico *was the presence of at least two reads with an alternative allele. The only significant explanatory variable for SNP reliability was the distance to the adjacent SNP site (Additional file 4), corroborating previous results. However the allele frequencies at SNP sites in the EST contigs (*in silico *MAF) and distance to the adjacent SNP site are not reliable predictors for SNP polymorphism. Interestingly, however, upon reviewing our SNP mining pipeline, we noticed that besides the final SNP calling by PolyBayes, an earlier SNP prediction is performed by MIRA, the EST clustering program. We therefore set to investigate the relative performance of both SNP calling approaches for the 696 genome-wide SNPs, reminding that all SNPs tested in the genotyping assay were predicted by PolyBayes with ≥99% probability. However, not all SNPs were tagged as such by MIRA. This inconsistency suggested the possible presence of SNP miscalls, which can be consequential to assay polymorphism. Out of the 696 SNPs considered, 632 were deemed reliable for *E. grandis *(GeneCall50 ≥ 0.4) and were divided in two classes: *in silico *SNPs tagged (348) and not tagged (284) by MIRA. When SNPs in these two classes were classified for polymorphism, a clear trend emerged indicating that a significantly larger proportion of SNPs called *in silico *by both PolyBayes and MIRA were in fact polymorphic in the GGGT assay when compared by those called exclusively by PolyBayes (p = 0.00024). Considering SNPs predicted only with PolyBayes the conversion rate was 43.4%. For SNPs called by both PolyBayes and MIRA the conversion rate was 58.1%. This result could be explained by the way that the two SNP calling algorithms operate. Polybayes likely suffered from the relatively large number of ESTs obtained in GeneBank for which no base quality values were available. In these cases an arbitrary Q value of 15 was assigned, a procedure that later impacted the estimate of SNP probability. MIRA, on the other hand, uses a sliding window of sequence quality instead of a single column, a strategy that favors the estimation of base quality and consequently an enhanced accuracy in SNP detection. This unexpected result suggests that in future SNP development efforts SNP tagging by MIRA could lead to higher SNP conversion rates.

### SNPs are reliable across *Eucalyptus *species and subgenera

Very few studies to date assessed the transferability of the same SNP genotyping array across a wide range of species within the same genus. In grape, transferability of SNPs assayed by the SNPlex™ Genotyping System (Applied Biosystems Inc.) averaged 18.8% across *V. vinifera *wild forms and only 2.3% when genotyping non-vinifera *Vitis *species. Only 4 SNPs out of 137 were polymorphic (MAF values ≥ 0.30), in non-vinifera *Vitis *species [35]. In the genus *Picea *in 279 resequenced genes that had at least one SNP in each of white spruce (*P. glauca*) and black spruce (*P. mariana*), only 4.7% of the observed SNPs were shared between the two species, requiring the development of separate 768 SNP arrays for each species. Recently, NGS of reduced representation libraries from 10 cultivated *V. vinifera *varieties and 6 wild *Vitis *species was used to develop a selected set of 8,898 SNPs and 24.3% of the were shared between *V. vinifera *and wild *Vitis *species [13]. In bovines when the 50K BovineSNP50 assay was applied to a set of DNA samples from six other species within Bovinae, including two from different genera, over 96% of the SNP produced genotype calls for at least five of the species including the four species within the genus *Bos *but only between 1 and 5% of the SNP that produced genotype calls were polymorphic despite the relatively recent divergence (1-5 Mya) between these species and *Bos taurus *[50]. In our study SNP genotyping reliability rates were high across nine species belonging to three sections within subgenus Symphomyrtus with more that 83% of the SNPs with call rates ≥ 95% and GeneCall50 ≥ 0.4 between 92% and 95%. Transferability rates were still satisfactory when going across subgenera with over 50% of the SNPs showing call rates ≥ 95% in two more distant species (Table 5). Estimates of divergence times among eucalypt lineages are still controversial. Those based on climatic and tectonic events suggest that the radiation of species within sections (<2 Mya) and sections within subgenus Symphyomyrtus (5-10 Mya) [54] are much more recent than estimates from molecular dating (5-10 Mya) and (13-36 Mya) respectively [55]. The relatively high transferability rates of our SNP panel across sections of Symphyomyrtus and even across distinct subgenera most likely agrees with a more recent species divergence and/or the maintenance of large population sizes over time and suggests that a reasonable proportion of SNPs developed for species of Symphyomyrtus pre-date the divergence from other subgenera and could be useful for genetic analysis in more distant species of *Eucalyptus*.

### Conclusion and perspectives

We have shown that a large number of SNPs assayed by the GGGT can be successfully developed from *in silico *sequence resources for a complex genome with very high nucleotide diversity. Using a systematic approach we have also shown that the application of stringent criteria on SNP flanking sequences *in silico *provides enhanced SNP reliability and good conversion rates across multiple species despite the absence of several of them in the EST collection used for SNP discovery. Although this might be a distinctiveness of *Eucalyptus*, where species radiation is probably recent, this study suggests that the Golden Gate assay could be a practical SNP genotyping method to carry out SNP-based population genomics studies in other outbreeding tropical tree genera. Nevertheless, SNP conversion success will be strongly dependent on having a representative and deep collection of sequences of the target species and a robustly selective SNP discovery pipeline including a moderate quality draft genome sequence now within reach for many species using third-generation single-molecule real-time DNA sequencing [56].

This study has provided the groundwork for a larger scale effort to develop a significantly larger SNP array for *Eucalyptus*. Following the successful reports published for a number of species to date [2,13,50], we are now using deep sequencing of reduced representation libraries (RRLs) to develop a large numbers of informative SNPs across the main species of *Eucalyptus *planted worldwide. SNPs discovered in diverse RRLs will provide exceptional opportunities to discover ancestry informative SNPs for genome-wide phylogenetic reconstructions and dating at different levels in *Eucalyptus *and related genera. Finally, a higher density SNP platform could be instrumental to implement Genomic Selection in *Eucalyptus *[57] although datapoint costs have to drop by an order of magnitude to become economically viable in tree breeding programs that encompass tens of thousands of samples. The current DArT marker array provides adequate density and genome-coverage at unbeatable costs [46]. However co-dominant SNPs would provide an added advantage to estimate haplotypes and allow the inclusion of non-additive effects in the predictive models, thereby increasing the expected accuracy of genomic predicted breeding values.

## Methods

### EST resources

Three EST datasets were used in this work. The first one is a Sanger 5' sequenced set of 101,240 ESTs from *E. grandis, E. globulus, E. pellita *and *E. urophylla *generated in the Genolyptus project [58]. The second one is a Sanger set of 34,801 sequences downloaded from dbEST that included 1,990 sequences from *E. grandis*, 19,860 from *E. gunnii*, 13,863 from *E. globulus *and the remaining from other species. The third was a dataset of 1,028,654 ESTs from NCBI SRA [accession SRX000427] generated from 21 individual trees of *E. grandis *using the 454 pyrosequencing technology [18]. These are the final numbers of sequences following removal of possible contaminations with vector, linker and adaptors using SeqClean and Crossmatch [59]. Poly (A/T) tails were trimmed, retaining a short 6-10 bp sequence to get quality ESTs for subsequent clustering, alignment and assembly. To assist in avoiding over-trimming of the hypothesized polyA/T site, an iterative scan for such homo-oligomers was implemented with est2assembly [60]. This routine also contributes to minimize errors produced by the 454 pyrosequencing methodology regarding detection of homopolymer sequences. User-supplied files with Phred scores [61] when available, were used as a measure of sequence quality in the pipeline. EST datasets downloaded from dbEST without associated quality information had their scores internally computed during the alignment process using Phrap [62].

### EST clustering and alignment

ESTs derived from each *Eucalyptus *species and with each technology (Sanger or 454) were grouped separately into clusters, each one expected to correspond to a single DNA segment and then aligned with the help of the user-supplied and internally computed base quality information using Phrap for Sanger reads or Newbler for 454 reads. Clustering was carried out using an algorithm based on the dissimilarity measures criteria with subword comparisons. Evaluation of the suitability of string dissimilarity measures for EST clustering was reported earlier [63]. The wcd EST clustering system [64] used in this study is an open source clustering system that provides efficient implementation of different dissimilarity measures, heuristics for speeding up clustering, a pre-clustering booster based on suffix arrays, as well as parallelized implementations based on MPI and Pthreads. The wcd EST clustering can be used to cluster large sets of mixed EST and RNA sequences, and is adaptable to shorter length error-prone sequencing technologies. For alignment, conservative Phrap parameters were applied to prevent misalignments in the resulting clusters and data quality information was used to ensure that maximum numbers of individual sequences were retained. A similar Phrap alignment investigation approach was used when processing sequences for loblolly pine SNP identification [65]. Clustering of the 454 sequences was carried out using Newbler 2.3 (Roche-applied.science.com). Aligned sequences in clusters comprised two or more sequences and could be a combination of one or more contigs. Sequences not aligned within a cluster corresponded to ESTs that were considered as unique within each dataset, i.e. singlets. Contigs and singlets were joined into one single FASTA file and another single file with quality data information was obtained. Each sequence in the FASTA file was identified in a third accompanying file which contained information about the *Eucalyptus *species of origin.

### EST assembly and identification of candidate SNPs

A general pipeline was put in place based on the software MIRA 2 - Whole Genome Shotgun and EST Sequence Assembler V2.9.25 with the enhanced 454 and Illumina/Solexa support. MIRA is an EST sequence assembler that specializes in reconstruction of pristine mRNA transcripts, while at the same time detecting and classifying single nucleotide polymorphisms (SNPs) occurring in different variations thereof [66]. MIRA combines a redundancy based approach with a symbolic pattern analyzer developed for recognition of column discrepancies in sequence alignments to allow detection of SNPs. In the algorithm implemented by MIRA the decision on whether discrepancies between similar EST sequences are significant or not relies more on the underlying quality information data. Under those circumstances, even a discrepancy caused by a single base in a single column of an alignment can be seen as a hint for a SNP site, i.e., if the base probability values of the bases in the immediate area are high and do not allow an alternative sequencing error hypothesis. The most important criterion adjudicating a SNP was therefore the group qualities of the bases in the immediate area of the SNP site - a SNP block - that can be calculated for different bases in a column. To investigate if group qualities of the bases in the vicinity of the SNP site could serve as a good predictor of true SNPs, SNPs from each one of those tags together with other non-tagged sites were selected from the list generated by MIRA. The selected predictors representing this classifier are a combination of two measures provided by the MIRA Assembler at the SNP site: the major allele score and the minor allele score. The minor allele is the allele occurring in the minority of the sequences at the SNP site, while the other is called the major allele. All the tagged or non-tagged SNP sites from the previous assembly step were submitted to an *in silico *evaluation for assessing the probability that a given site was polymorphic. SNP prediction was carried out using PolyBayes version 3.0 [67] with a p prior of 0.01 corresponding to an expected mean frequency of one SNP every 100 nucleotides and putative SNPs called with a stringent cutoff probability of P_SNP_≥ 0.99.

### SNP selection for GGGT assay design

Following the Polybayes probability and SNP vicinity scores provided by the alignment, a set of parameters were defined to construct filters to select SNPs for the GGGT assay design. Initially all SNPs had to have at least 60 bases available on each SNP side to allow designing allele and locus specific oligos for the GGGT assay. Additionally the following parameters were used: (1) the *in silico *estimated minor allele frequency (MAF) at the SNP site; (2) the number of EST reads by target species aligned at each SNP site; and (3) the presence of additional SNPs along the SNP flanking sequences. The impact of these parameters on SNP reliability and polymorphism validated by the GGGT assay was evaluated by sequentially implementing increasingly stringent *in silico *SNP filtering levels as follows:

#### Filter F0

bi-allelic SNPs with ≥ 5 reads on SNP position, and a minimum of one read with the alternative base;

#### Filter F1

bi-allelic SNPs with ≥ 5 reads on SNP position and a Minor Allele Frequency (MAF) ≥ 0.2;

#### Filter F2

same as F1 plus a minimum of one read derived from *E. globulus *or *E. gunnii*; these species belong to the section *Maidenaria*, phylogenetically separate from *E. grandis, E. urophylla *and *E. pellita *that belong to section *Latoangulatae*;

#### Filter F3

same as F2 plus a minimum of 100 bases on each SNP side without repetitive elements and a minimum of 20 bases flanking the SNP on each side without any additional SNPs in the contig;

#### Filter F4

same as F3 but increasing to a minimum of 60 bases flanking the SNP on each side without any additional SNPs in the contig.

As the SNPs were derived from cDNA sequences, the occurrence of intron/exon border in the flanking sequences upon which the oligos are later designed, may cause significant genotyping failure [51]. To mitigate this problem an additional analysis based on the Exonerate program [68] using the est2genome model, was implemented on all SNPs that passed one or more of the five filters. Only SNPs that had 30 bp on each side belonging to the same exon were selected. The 4.5X draft genomic assembly of *Eucalyptus grandis *used as reference was downloaded from the EUCAGEN (*Eucalyptus *Genome Network) site at http://Eucalyptusdb.bi.up.ac.za.

From each one of the five filtered subsets of SNPs a random sample between ~300 and 600 SNPs was derived to be subsequently submitted to the Assay Design Tool (ADT) made available by Illumina with a target of having between 96 and 288 SNPs to be effectively designed and validated. Each SNP receives a SNP score that is a predictor of the likelihood of genotyping success using the GGGT assay. ADT generates scores for each SNP that could vary from 0 to 1. According to the Illumina standard recommendations, a SNP with score ≥ 0.6 has a high probability to be converted into a successful genotyping assay. Based on the ADT scores returned by Illumina, a subset of SNPs to be included in the SNP bead array was randomly selected from the final set of SNPs obtained with a more stringent ADT ≥ 0.8 for each one of the five filter treatments.

### SNPs selected in candidate genes

A list of 42 genes described in the literature as being putatively associated with relevant wood phenotypes in *Eucalyptus *was compiled. These were genes involved in lignin biosynthesis [69,70], genes derived from expression studies of wood formation using microarrays [71,72], expression-QTL mapping [73,74] and association studies [48]. GenBank accession numbers for these genes or their names as described in the respective papers were used to select and assemble ESTs from the databases for SNPs discovery. All SNPs that satisfied the standard requirement of 60 bases of available sequence on each SNP side and at least two reads with alternative bases at the SNP position (i.e a more relaxed selection than Filter F0 which required ≥ 5 reads at the SNP position) were submitted to the Illumina assay design tool and those with ADT score ≥ 0.6 were subsequently selected to populate the bead pool array.

### Plant material and DNA extraction

Population samples of unrelated trees of each one of the five most widely planted and bred species worldwide were used for SNP reliability, inter-specific transferability and polymorphism assessment. All these five species belong to the same subgenus *Symphomyrtus *but to different sections. Sample sizes were N = 24 for *E. grandis *(section *Latoangulatae*), N = 24 for *E. globulus *(section *Maidenaria*), N = 16 for *E. urophylla *(section *Latoangulatae*), N = 16 for *E. camaldulensis *(section *Exsertaria*) and N = 16 for *E. nitens *(section *Maidenaria*), totaling 96 individuals. For *E. grandis*, twelve trees from each of two different provenances were sampled, Atherton (17°15'S 145°28'E) and Coffs Harbor (30°18'S 153°07'E); for *E. globulus *twelve trees were from Jeeralang (38°24'S 146°28'E) and twelve from Flinders Island (40°00'S 148°07'E); for *E. urophylla *all trees were from Flores Island (8°39'S 122°15'E), for *E. camaldulensis *all trees were from Walsh River (17°17'S 144°88'E) and for *E. nitens *trees were from Eastern Ebor (30°24'S 152°29'E). Small samples of N = 8 individuals were also genotyped for other six species of more limited world relevance to provide a preliminary assessment of SNP transferability across a wider inter-specific range and across two additional subgenera. These were: *E. dunnii, E. saligna, E. tereticornis*, these four also belonging to the subgenus *Symphomyrtus*, and *E. cloeziana *and *E. pilularis *belonging to two different subgenera, *Idiogenes *and *Eucalyptus*, respectively. DNA extractions from fresh expanded leaves were carried out as described earlier using a modified CTAB procedure [75] and quantified using the PicoGreen assay (Invitrogen, Carlsbad, CA, USA) in a Nanodrop 3300 micro-volume fluorospectrometer and standardized to Illumina-specified concentrations for SNP genotyping (50-100 ng/μL).

### SNP genotyping

Selected SNPs were used to construct an Illumina bead array of 768 SNPs based on the GoldenGate assay (Illumina Inc., San Diego, California). Out of these 768 SNPs, 696 were distributed across the five SNP selection filters while 72 SNPs were derived from specific candidate genes. Genotyping was performed using an Illumina BeadStation 500 GX (Illumina, San Diego, CA) at the Genome facility of the Interdisciplinary Center for Biotechnology Research (ICBR) of the University of Florida using the protocol described earlier [76]. SNP data were analyzed using GenomeStudio V2009.2 Genotyping module 1.5.16 (Illumina, San Diego, CA) that clusters and calls the data automatically, allowing visualization of the data directly for downstream analysis. Call rates, GeneTrain and GenCall scores were exported using the GenomeStudio internal Locus Summary Report Tool for the subsequent analysis of SNP reliability and polymorphism.

### SNP genotyping reliability

SNP reliability was evaluated by the GeneTrain and the GeneCall scores. The GenTrain score, a statistics with a value between 0 and 1, was estimated for each SNP to assess the quality of the shapes of the genotype clusters (homozygous and heterozygous) and their relative distance to each other. A GenCall score, estimated for each datapoint (SNP × individual sample), is designed to rank particular DNA samples or SNP loci and is obtained by the product of the GenTrain Score and a data-to-Bayesian-model fit score as implemented by the Genome Studio software. Genotypes with lower GenCall scores are located further from the center of the genotype cluster and have a lower reliability. To assess the reliability of each individual SNP the GenCall scores for all typed samples for each SNP were used to estimate a GeneCall50 (GC50) score that corresponds to the 50th percentile (median) of the distribution of the GenCall scores for that SNP. A GeneCall50 score threshold ≥ 0.40 was used to declare a SNP reliable. SNPs genotyping performance was additionally assessed by the call rate for each SNP using a GeneCall score cutoff ≥ 0.25 for each datapoint (SNP × Individual sample) following the Illumina recommended threshold for GGGT [76]. Reliability parameters were evaluated consolidating genotyping data for all species that had 16 or more individuals genotyped and for each species separately.

### SNP polymorphism information content

Polymorphism was evaluated by the conventional MAF (Minimum Allele Frequency) parameter. A SNP was considered polymorphic if MAF ≥ 0.05. Polymorphism was evaluated consolidating data for all samples together, irrespective of species, and also for each species separately but only for those species that had at least 16 samples (32 chromosomes) analyzed. Species for which only eight individuals were analyzed could not provide acceptable estimates of polymorphism level.

### Assessment of the *in silico *variables on SNP reliability and polymorphism

Goodness-of-fit for normality tests were carried out on GeneTrain and GeneCall50 scores estimated for each SNP. Mann-Whitney non-parametric tests were carried out to assess the impact of the SNP filtering levels (F0 through F4) on GeneTrain and GeneCall50 scores. Furthermore, using a GeneTrain score ≥ 0.4, a GC50 score ≥ 0.4, a call rate ≥ 95% as thresholds for declaring a reliable SNP and a MAF ≥ 0.05 as a threshold to declare a polymorphic SNP, pair-wise Chi-square Pearson contingency tests were used to assess the impact of the five filtering levels on the final proportions of SNPs declared as being reliable and polymorphic. The final SNP conversion rate estimated by the proportion of polymorphic SNPs (MAF ≥ 0.05) within the ones deemed reliable (GeneTrain score ≥ 0.4 and GeneCall50 score ≥ 0.4) was estimated for the whole data set and for each one of the main five species separately.

### Inheritance-based SNP validation

Heritability of SNP allelic transmission was calculated for each SNP using data from a total of 48 parent-parent-offspring trios involving different *Eucalyptus *species as parents. Both parents and 24 offspring individuals were randomly selected from each one of two segregating populations, one derived from a *E. grandis × E. urophylla *interspecific cross and the second one from a four-species cross involving a *E. dunni × E. grandis *male parent and a *E. urophylla × E. globulus *female parent. Heritability was estimated using Genome Studio internal heritability report tool by counting the proportion of correct allelic transmissions for each set of 24 trios.

### Sequence-based validation of SNP genotypes

Genotypes for queried SNPs were validated for a sample of *E. camaldulensis *by comparing the GoldenGate called genotypes with NGS based genotypes derived from shotgun genomic reads (Illumina 2 × 76 bases paired-end reads) providing an estimated 2X coverage of the 630 Mbp *Eucalyptus *genome. Sequence based genotypes were called only for SNP positions that had at least five clustered reads on the reference genome and sequence quality Q ≥ 20. A sample size of 5 reads provides an expected probability of 0.9375 of detecting at least one read with the alternative allele if the genotype is heterozygous. Sequence based genotypes were declared based on a simple binomial test where a null hypothesis of an expected 1:1 ratio of the read counts was set. When the null hypothesis was not rejected a heterozygous genotype was declared. Rejection of the null hypothesis, on the other hand, led to the inference of a homozygous genotype at the SNP.

## Authors' contributions

DG was responsible for project conception, statistical analyses, manuscript writing, overall supervision of project execution and funding; OSJr was involved in the conception and development of the SNP discovery and design pipeline, and contributed to statistical analyses and manuscript preparation; MK contributed to SNP genotyping; BML contributed to DNA preparation; DAF was responsible for candidate gene selection and helped with data analysis; GJPJr conceived the bioinformatics pipeline development and was responsible for overall bioinformatics supervision. All authors have read and approved the final manuscript.

## Supplementary Material

Additional file 1 **Information on the 768 *Eucalyptus *SNPs developed**. SNPs are organized in two separate lists, one with the 696 genome-wide SNPs selected with the five *in silico *sequential filtering levels (locus names start with F0 through F4 - see methods) and a second one with the 72 candidate gene SNPs. Information on the SNP type, SNP flanking sequences used for GGGT assay design, ADT score, Call rate, Minor allele frequency, GeneTrain score and GeneCall50 score is provided, estimated from the 96 DNA samples typed of the five main Eucalyptus species (*E. grandis, E. urophylla, E. globulus, E. nitens *and *E. camaldulensis*). Contig sequence used for SNP discovery and BLAST annotation are also provided. Click here for file

Additional file 2 **Supplementary material S2**. Counts and percentages of polymorphic SNPs (MAF ≥ 0.05) from a total of 711 reliable SNPs in all 26 combinations of the five main Eucalyptus species surveyed. Click here for file

Additional file 3 **Supplementary material S3**. Results of the NGS-based validation of SNP genotypes. Golden Gate Genotype calls (GGGT genotype) for a *Eucalyptus camaldulensis *individual were compared to sequence-based genotypes inferred from Illumina short read sequencing based on a binomial test where a null hypothesis of an expected 1:1 ratio of the read counts was set. When the null hypothesis was not rejected a heterozygous genotype was declared. Rejection of the null hypothesis, on the other hand, led to the inference of a homozygous genotype at the SNP. Shaded in grey are the seven SNPs genotypes that showed divergent results between GGGT and NGS genotype calls. Click here for file

Additional file 4 **Supplementary material S4**. Results of logistic regression of the in silico variables used in the SNP discovery and filtering pipeline in *E. grandis *on SNP Reliability and SNP Polymorphism treated as binary characters (reliability defined by GeneCall50 ≥ 0.4 and polymorphism by MAF ≥ 0.05). Click here for file
