# Supplementary material for: High-throughput SNP genotyping in the highly heterozygous genome of Eucalyptus: assay success, polymorphism and transferability across species
Source: BMC Plant Biol. 2011 Apr 14;11:65. doi: 10.1186/1471-2229-11-65 (PMC3090336; doi:10.1186/1471-2229-11-65)
Supplement: Additional file 2 — Supplementary material S2. Counts and percentages of polymorphic SNPs (MAF ≥ 0.05) from a total of 711 reliable SNPs in all 26 combinations of the five main Eucalyptus species surveyed. [file 1471-2229-11-65-S2.PDF]

Supplementary material S2. Counts and percentages of polymorphic SNPs (MAF ≥ 0.05) from a total of 711 reliable SNPs in all 26 combinations of the five main *Eucalyptus* species surveyed.

**Species combination**	**Number of polymorphic SNPs**	**(%)**
E. grandis + E. urophylla	209	29.4
E. grandis + E. globulus	117	16.5
E. grandis + E. nitens	128	18.0
E. grandis + E. camaldulensis	194	27.3
E. urophylla + E. globulus	107	15.0
E. urophylla + E. nitens	120	16.9
E. urophylla + E. camaldulensis	187	26.3
E. globulus + E. nitens	104	14.6
E. globulus + E. camaldulensis	118	16.6
E. nitens + E. camaldulensis	127	17.9
E. grandis + E. urophylla + E. globulus	89	12.5
E. grandis + E. urophylla + E. nitens	90	12.7
E. grandis + E. urophylla + E. camaldulensis	144	20.3
E. urophylla + E. globulus + E. nitens	77	10.8
E. urophylla + E. globulus + E. camaldulensis	90	12.7
E. globulus + E. nitens + E. camaldulensis	78	11.0
E. globulus + E. nitens + E. grandis	81	11.4
E. nitens + E. camaldulensis + E. grandis	95	13.4
E. nitens + E. camaldulensis + E. urophylla	94	13.2
E. camaldulensis + E. grandis + E. globulus	94	13.2
E. grandis + E. urophylla + E. globulus + E. nitens	64	9.0

E. grandis + E. urophylla + E. globulus + E. camaldulensis	78	11.0
E. urophylla + E. globulus + E. nitens + E. camaldulensis	64	9.0
E. globulus + E. nitens + E. camaldulensis + E. grandis	64	9.0
E. nitens + E. camaldulensis + E. grandis + E. urophylla	75	10.5
E. grandis + E. urophylla + E. globulus + E. nitens + E. camaldulensis	55	7.7
