# Supplementary material for: High-throughput SNP genotyping in the highly heterozygous genome of Eucalyptus: assay success, polymorphism and transferability across species
Source: BMC Plant Biol. 2011 Apr 14;11:65. doi: 10.1186/1471-2229-11-65 (PMC3090336; doi:10.1186/1471-2229-11-65)
Supplement: Additional file 3 — Supplementary material S3. Results of the NGS-based validation of SNP genotypes. Golden Gate Genotype calls (GGGT genotype) for a Eucalyptus camaldulensis individual were compared to sequence-based genotypes inferred from Illumina short read sequencing based on a binomial test where a null hypothesis of an expected 1:1 ratio of the read counts was set. When the null hypothesis was not rejected a heterozygous genotype was declared. Rejection of the null hypothesis, on the other hand, led to the inference of a homozygous genotype at the SNP. Shaded in grey are the seven SNPs genotypes that showed divergent results between GGGT and NGS genotype calls. [file 1471-2229-11-65-S3.PDF]

Supplementary material S3. Results of the NGS-based validation of SNP genotypes. Golden Gate Genotype calls (GGGT genotype) for a *Eucalyptus camaldulensis* individual were compared to sequence-based genotypes inferred from Illumina short read sequencing based on a binomial test where a null hypothesis of an expected 1:1 ratio of the read counts was set. When the null hypothesis was not rejected a heterozygous genotype was declared. Rejection of the null hypothesis, on the other hand, led to the inference of a homozygous genotype at the SNP. Shaded in grey are the seven SNPs genotypes that showed divergent results between GGGT and NGS genotype calls.

SNP	GGGT genotype	GeneCall score	# reads of allele 1	# reads of allele 2	p value of the binomial test	Sequence-based genotype
F0EU1906AG187	A/A	0.6	0	9	0.004	A/A
F0EU3122AG650	G/G	0.6	0	5	0.063	G/G
F0EU4800CT651	T/T	0.6	0	7	0.016	T/T
F0EU5748CT554	C/T	0.5	2	6	0.289	C/T
F0EU6977AG438	A/A	0.6	0	20	0.000	A/A
F0EU7320CT154	T/T	0.6	0	5	0.063	T/T
F1EU13828CT303	C/C	0.8	0	6	0.031	C/C
F1EU20733CG455	C/G	0.4	5	4	1.000	C/G
F1EU6809AC458	A/A	0.8	0	6	0.031	A/A
F2EU10266CT140	C/T	0.5	5	2	0.453	C/T
F2EU1058CT822	T/T	0.7	0	7	0.016	T/T
F2EU1069CG708	C/C	0.8	0	6	0.031	C/C
F2EU1136CT406	C/C	0.8	0	5	0.063	C/C
F2EU1313AT656	T/T	0.5	2	11	0.022	T/T
F2EU2014AT1270	T/T	0.8	0	6	0.031	T/T
F2EU2683AT1067	A/T	0.8	0	6	0.031	T/T
F2EU3019AC984	C/C	0.9	0	5	0.063	C/C
F2EU3250AG736	G/G	0.5	0	6	0.031	G/G

F2EU4400GT662	G/G	0.5	1	8	0.039	G/G
F2EU4446CT914	C/C	0.4	16	14	0.856	C/T
F3EU4306CT335	C/C	0.5	0	7	0.016	C/C
F3EU4936GT99	G/G	0.6	0	5	0.063	G/G
F3EU6062AG185	G/G	0.5	7	2	0.180	A/G
F3EU6733AT361	A/A	0.7	3	3	1.000	A/T
F3EU8621GT658	G/G	0.6	0	7	0.016	G/G
F3EU9807AG208	A/A	0.7	0	7	0.016	A/A
F4EU3516AG1124	A/A	0.8	0	5	0.063	A/A
F4EU3618AC842	A/C	0.7	3	2	1.000	A/C
F4EU3652AG696	A/A	0.9	0	6	0.031	A/A
F4EU4003AG206	A/A	0.6	0	5	0.063	A/A
F4EU4154CT892	C/C	0.9	0	9	0.004	C/C
F4EU4604AG115	G/G	0.8	0	8	0.008	G/G
F4EU4705CT962	T/T	0.6	0	5	0.063	T/T
F4EU4713GT726	G/G	0.5	8	8	1.000	G/T
F4EU4945CT587	T/T	0.5	0	8	0.008	T/T
F4EU5213AT538	A/A	0.6	0	10	0.002	A/A
F4EU5522AT579	A/A	0.8	0	7	0.016	A/A
F4EU5683CT556	C/C	0.5	0	5	0.063	C/C
F4EU6143CT315	T/T	0.6	0	5	0.063	T/T
F4EU6853AG467	A/G	0.8	4	3	1.000	A/G
F4EU6992AG425	A/A	0.6	0	5	0.063	A/A
F4EU7089AG422	A/G	0.7	0	5	0.063	A/A
F4EU7155AG603	A/A	0.6	0	5	0.063	A/A
F4EU8042CT444	T/T	0.7	2	3	1.000	C/T
F4EU8069AG315	A/A	0.8	0	5	0.063	A/A
F4EU8200CT140	T/T	0.6	0	7	0.016	T/T
F4EU8305AG803	A/G	0.5	2	5	0.453	A/G
F4EU8396CT780	C/C	0.6	0	5	0.063	C/C

F4EU859CT829	T/T	0.5	13	2	0.007	T/T
F4EU864GT1617	G/T	0.4	3	2	1.000	G/T
