# Supplementary material for: High-throughput SNP genotyping in the highly heterozygous genome of Eucalyptus: assay success, polymorphism and transferability across species
Source: BMC Plant Biol. 2011 Apr 14;11:65. doi: 10.1186/1471-2229-11-65 (PMC3090336; doi:10.1186/1471-2229-11-65)
Supplement: Additional file 4 — Supplementary material S4. Results of logistic regression of the in silico variables used in the SNP discovery and filtering pipeline in E. grandis on SNP Reliability and SNP Polymorphism treated as binary characters (reliability defined by GeneCall50 ≥ 0.4 and polymorphism by MAF ≥ 0.05). [file 1471-2229-11-65-S4.PDF]

Supplementary material S4. Results of logistic regression of the *in silico* variables used in the SNP discovery and filtering pipeline in *E. grandis* on SNP Reliability and SNP Polymorphism treated as binary characters (reliability defined by GeneCall50 ≥ 0.4 and polymorphism by MAF ≥ 0.05).

***In silico* variable**	**SNP Reliability**		**SNP Polymorphism**	
	Z	p value	Z	p value
In silico estimated MAF	0.585	0.558	-0.994	0.320
# EST reads of the species at the SNP site	-1.299	0.194	-0.224	0.823
Highest quality of the minor allele base	0.475	0.635	1.347	0.178
Highest quality of the major allele base	1.437	0.151	-2.903	0.004
Minimal distance to the next SNP site	2.681	0.007	1.028	0.304
